# Adult sacrococcygeal teratoma: A review

**DOI:** 10.1097/MD.0000000000032410

**Published:** 2022-12-30

**Authors:** Jia-Xing Guo, Jian-Guo Zhao, Ying-Na Bao

**Affiliations:** a Department of Radiotherapy, The Affiliated Hospital of Inner Mongolia Medical University, Hohhot, China.

**Keywords:** sacrococcygeal teratoma (SCT), teratoma, therapy

## Abstract

Sacrococcygeal teratomas (SCT) in adults are extremely rare, and most SCTs are located either mainly outside the pelvis, with a small number of intrapelvic components, or mostly in the pelvis (types III and IV). The etiology of teratomas remains unknown. Most teratomas are benign, and approximately 1 to 2% of teratomas undergo malignant transformation, including squamous cell carcinoma, adenocarcinoma, sarcoma, and other malignancies. Most SCTs grow insidiously, and their symptoms are not easily detected in the early stages. Some cases may only be discovered through physical examination or compression symptoms when the tumor reaches a detectable size. Computed tomography and magnetic resonance imaging have high detection rates for presacral space-occupying lesions and can provide imaging details with guiding significance for the selection of surgical methods. Surgical resection is the preferred treatment option for SCT and can determine the pathological type. Common sacrococcygeal malignancies are mainly immature teratomas and mature teratomas. When the presence of malignant components is confirmed, the treatment model should be adjusted according to pathological type.

## 1. Introduction

A teratoma is a common germ cell tumor that is mainly composed of totipotent stem cells. Pluripotent stem cells are concentrated in the Hensen node in front of the sacrococcygeal bone. Therefore, the sacrococcygeal region is a common extra-gonadal site. Adult sacrococcygeal teratomas (SCT) are extremely rare, with an incidence of 1 in 40,000 to 1 in 63,000 patients and a female to male ratio of 3:1.^[1]^ In 1973, Altman compiled survey reports from the American Department of Paediatric Surgery. SCT is classified into 4 types according to tumor location^[2]^: type I (46%), completely outside the pelvis; type II (35%), mainly outside the pelvis, but with a small number of intrapelvic components; type III (9%), mostly in the pelvis, with a small amount of external mass; and type IV (10%), completely in the pelvis. Type I and II tumors account for 80% of all SCTs and are the most common in neonates. These tumors can be diagnosed and resected at early stages. Most adult SCTs are type III or IV tumors.

### 1.1. Etiology and pathological types of teratoma

The etiology of teratomas remains unknown. Teratomas are thought to originate from totipotent stem cells, which are usually present in the ovaries and testes, and are sometimes abnormally present in isolated midline embryos. SCTs are thought to arise from migration of totipotent stem cells to the coccygeal region.^[3]^ Other hypotheses for the origin of teratomas include the asexual reproduction of germ cells in intragonadal or extragonadal sites; “wandering” germ cells of non-parthenogenetic origin left during the migration of embryonic germ cells from the yolk sac to the gonad; origin from other totipotent embryonic cells^[4,5]^; and the result of a twinning attempt.^[6]^ Familial presacral teratomas are inherited in an autosomal dominant pattern.^[7]^

Teratomas can be classified as mature, immature, or malignant. Most teratomas are benign, and approximately 1 to 2% of teratomas can undergo malignant transformation into squamous cell carcinoma, adenocarcinoma, sarcoma, and other malignancies.^[8]^ Mature teratomas are also known as dermoid cysts. Most teratomas are cystic and contain mature tissues from the ectoderm (such as skin, hair follicles, and cortical glands), mature tissues from mesoderm (such as muscle tissue and urinary tissue), and mature tissues of endoderm (such as lung and gastrointestinal tissue).^[9]^ In rare cases, teratomas may be solid tumors composed of a variety of heterogeneous tissues and organizational structures that appear benign. These structures were derived from all 3 germ layers. The mechanism of formation may be related to failure of the second meiotic division or failure of the first meiotic division of premeiotic cells.^[10]^ The incidence of malignant transformation of mature cystic teratoma is 0.2 to 2%.^[11,12]^ Immature teratomas are also called malignant, teratoblastomas, or embryonal teratomas.

### 1.2. Symptoms of SCT

Most SCTs grow insidiously, and their symptoms are not easily detected at an early stage. When the tumor reaches a certain size, it may be discovered through physical examination or compression symptoms.^[13]^ Intrapelvic masses are common in adults,^[13]^ and compression symptoms are site-related and may present with constipation, sacrococcygeal pain, bladder dysfunction, lower extremity venous congestion, and neurological symptoms. SCT may be associated with infection, manifesting as an ulcer in the sacrococcygeal region, which needs to be differentiated from an anal fistula.^[14]^ In addition, previous studies suggest that digital rectal examination can be used to assess tumor size, texture, and extent of invasion, which can help differentiate SCT from anal fistula.^[15]^

### 1.3. Medical examination of SCT

Owing to its anatomical characteristics, SCT biopsy is difficult to perform. Furthermore, the internal tissues of teratomas are diverse, and SCT biopsy is difficult to evaluate. When malignancy is suspected, preoperative percutaneous or transrectal biopsy should be avoided due to the risk of recurrence or metastasis. Ultrasound is a widely used, cost-effective, and safe method, and an important modality for routine screening. A retrospective analysis of 47 cases of teratoma confirmed by surgery and pathology showed that preoperative transrectal ultrasonography revealed that multilocular cystic and mixed cystic solids were more common on transrectal ultrasonography. Malignant tumors are mostly solid or mixed cysts and masses. Color Doppler ultrasonography revealed that the blood flow in solid malignant SCTs is rich, but the blood flow signal in rapidly growing SCTs can also be rich, and arteriovenous fistulas are rarely seen. Different degrees of blood flow signals can also be seen in solid components rich in nerve tissues. Therefore, the accurate identification of benign and malignant adult teratomas by color Doppler ultrasound needs to be verified in a large sample of cases.^[16]^

Computed tomography (CT) and magnetic resonance imaging (MRI) have a high detection rate for presacral space-occupying lesions, and these modalities can evaluate the relationship between space-occupying lesions and the rectum and sacrococcygeus, which has a certain guiding significance for the selection of surgical methods. MRI is more specific and accurate than CT for assessing the extent of soft tissue in SCT. Sagar et al demonstrated the ability of MRI to differentiate between benign and malignant tumors with a specificity and sensitivity of 97% and 88%, respectively. The corresponding preoperative biopsy rates were 100% and 83%, respectively.^[17]^ SCT contain varying proportions of soft tissue, fat, calcification, and fluid components. CT can detect fat and calcification, and the characteristic CT values of adipose tissue and calcium are −80 to −20 HU and 80 to 300 HU, respectively, which are easy to distinguish. CT scans are very sensitive in revealing calcification in >50% of malignant tumors. However, other studies have reported calcifications in both benign and malignant teratomas. Therefore, tumor calcification cannot be used as a criterion to distinguish between benign and malignant tumors.^[18]^

Typical mature teratomas are mostly cystic and fatty, with few parenchyma.^[19]^ On MRI, a teratoma is a round or irregularly separated cystic mass with a uniform signal in the cyst. The signal on T1WI and T2WI is high, and a signal reduction area can be seen in the mass on T2WI of the fat-suppressed sequence. Teratomas usually contain fat components, and the intratumoral fat signal is significantly reduced on fat-suppression sequence images, which is a characteristic manifestation of teratomas.^[20]^

Giant teratomas compress the pelvic cavity and pelvic floor structure, occupy most of the pelvic cavity, and protrude to the tail or to the abdominal cavity and tail, compressing the rectum and uterus to move forward, and the anus to move forward and downward. If the solid part of the teratoma is >50% of the tumor body and the boundary with the surrounding tissue is unclear, it is often suggestive of malignant transformation.^[20]^

Tumor markers are substances that are characteristically present in tumor cells, abnormally produced by tumor cells or produced by the host in response to tumor stimulation. Benign teratomas typically do not contain tumor markers. When the malignant pathological tissue in teratomas is mainly immature teratomas and malignant transformation of mature teratomas, related tumor markers may be increased. The tumor markers CA724, CA199, and carcinoembryonic antigen are highly recognizable in SCTs and are thus useful for diagnostic and prognostic evaluation.^[21]^ The CA199-positive rates in immature, mature, and malignant SCTs were 57%, 57%, and 48.6%, respectively.^[22]^ Elevated α-fetoprotein (AFP) levels are common in yolk sac tumors, certain immature teratomas, embryonal and polyembryoma carcinomas, and mixed germ cell tumors. In adult SCT, an AFP level >250 µg/mL indicates malignancy.^[23]^ A recent study showed that AFP has a sensitivity and specificity of 79% and 95%, respectively, for diagnosing recurrent SCT in children. However, these data were based on patients with elevated AFP levels and recurrence, and their sensitivity may be higher. When a teratoma contains different malignant components, including squamous cell carcinoma, basal cell carcinoma, adenocarcinoma, sarcoma, thyroid cancer, or yolk sac tumor, the corresponding tumor markers for each of these components should be monitored.

Differential diagnoses of SCT in adults include chordoma, meningocele, sacral giant cell tumor, pilonidal sinus, rectal duplication cyst or anal gland cyst, sacral osteomyelitis, perirectal abscess, fistula, granuloma, and tuberculosis.^[24]^

### 1.4. Treatment of SCT

Surgical resection is the preferred treatment option for patients with SCT. Three surgical approaches are primarily used: transabdominal, transsacral, and transabdominal-sacral. The transabdominal approach is usually used for higher lesions because it exposes the tumor to the back of the rectum. The transsacral approach is preferred for small tumors located in the lower pelvis. The transsacral approach is considered a good option for tumors ≤10 cm in diameter and located below the third sacral vertebra, although specific criteria have not been established.^[25]^ Recent reports have shown that a small number of SCTs can be safely resected laparoscopy,^[26,27]^ and some surgeons have also used endoscopy to remove tumors through the sacral approach.^[28]^ Resectability depends on the pathological type and extent of the tumor. Resection can involve portions of the gastrointestinal tract, kidneys, bladder, spleen, aorta, and vena cava.^[29]^ If the tumor is predominantly cystic, after exposure of the tumor through the sacrum, the cyst fluid can be extracted, allowing the tumor to collapse, and the tumor can then be completely resected using a posterior-only approach.^[15]^ However, malignant transformation may occur with incomplete or intralesional resections.

Whether coccyx should be removed during surgery is controversial.^[18]^ The main reasons for coccyx resection are based on the following considerations: for large tumors, exposure during surgery may be required; SCT may originate from pluripotent cells of the coccyx; and tumors may tend to attach to the coccyx, and resection of the coccyx can prevent relapse. Surgical outcomes of SCT in adults have not been extensively reported, and a standard treatment regimen for SCT remains undefined.^[30]^ Therefore, regardless of the method, complete resection should be the primary treatment goal based on individualization, and quality of life should be improved as much as possible.

Unlike teratomas at other sites, SCT often have no capsule or pseudocapsule, and complete resection of the lesion is difficult. The postoperative recurrence rate of lesions with incomplete resection is as high as 37%.^[31]^ Derikx et al^[32]^ reported 173 cases of SCT; among the 143 benign cases, 11 cases showed recurrence, and the average recurrence time was 10 months after the operation. Xiufeng et al reported 12 cases of SCT, and one of these cases in a female patient showed recurrence at 14 months after operation.^[33]^ Qiongqiong et al reported 5 cases of SCT, with 4 cases of recurrence after surgery, with an average recurrence time of 11 years.^[34]^ Immature and malignant histology, incomplete surgical resection, and failure of en bloc resection of the coccyx are associated with a high risk of recurrence.^[35]^ The overall recurrence rate was approximately 10% for mature teratomas and 20% for immature teratomas.^[36]^ Notably, the coccyx may contain foci of totipotent cells, and if the coccyx is not resected simultaneously, the risk of tumor recurrence is 30 to 40%.^[37]^

Common sacrococcygeal malignancies are mainly immature teratomas and mature teratomas. Upon confirmation of the presence of malignant components after postoperative pathology, the treatment mode should be adjusted according to the pathological type and appropriate chemotherapy and radiotherapy are required. Because the current understanding of adult presacral teratomas is limited, the adjuvant treatment model of ovarian malignant teratomas has been applied to provide diagnosis and treatment ideas. Platinum-based chemotherapy is currently the standard treatment regimen, with bleomycin, etoposide, and cisplatin as the preferred regimen. Although data are limited, studies have indicated that 3 cycles of adjuvant bleomycin, etoposide, and cisplatin therapy reduce relapse.^[38]^ In cases where the pathological type of ovarian malignancy is a mucinous tumor, the adjuvant chemotherapy regimen is 5-fluorouracil + leucovorin + oxaliplatin or capecitabine + oxaliplatin. Radiotherapy is another treatment method that has been previously reported. In a case report by Kumar et al, a 37-year-old man with SCT underwent surgical resection of the presacral mass and coccyx, and histopathology confirmed that the mature teratoma had transformed into adenocarcinoma. The patient received postoperative radiotherapy (45 Gy/1.8 Gy/25 F) and bleomycin combined with etoposide and cisplatin over 3 cycles of chemotherapy.^[39]^ Surgery is another treatment option for recurrent and malignant SCT. In another study, a patient with a pathologically diagnosed malignant SCT showed recurrence at 84 months of postoperative follow-up. After the second surgery, the patient had no recurrence within 6 months.^[15]^

The risk of malignant transformation in adult SCT ranges from 1 to 12%.^[22]^ Malignancy of any component may occur; however, ectodermal squamous cell carcinoma is the most common component.^[40]^ Others include basal cell carcinoma, melanoma, adenocarcinoma, sarcoma, and thyroid cancer. One report^[21]^ suggested that adult teratomas transform into mucinous carcinoma, a distinct subtype of mucinous adenocarcinoma that is more malignant than other mucinous adenocarcinomas. Mucinous carcinomas are surrounded by mucins, insensitive to chemoradiotherapy, and have a poor prognosis.^[41]^ This type of adenocarcinoma is characterized by rapid growth, invasion of the intestinal wall, and infiltration of surrounding organs.^[41]^ In a case of a malignant teratoma that had already metastasized at the time of discovery, postoperative biopsy of the anterior sacrum showed malignant transformation of the teratoma, and the histological type was squamous cell carcinoma. After postoperative radiotherapy and chemotherapy, the patient was followed up for 10 months and is still alive at the time of publication.^[23]^ Age, tumor parenchyma, and recurrent infections were associated with malignancy, but tumor size alone was not a factor. Metastases of malignant teratomas include lymphatic metastasis to the retroperitoneal lymph nodes, hematogenous metastasis to the lung and bone, and poor prognosis after distant metastasis.^[42]^

## 2. Conclusion

Owing to the increased malignancy rate of teratomas with patient age, early excision is recommended. Resection of benign SCT tumors results in a 5-year survival rate of approximately 100%. However, when SCT is transformed into carcinoma or sarcoma, the prognosis is poor. Teratomas that are not resected or are incompletely resected may lead to late recurrence (defined as recurrence after a recurrence-free interval of >2 years after the completion of primary treatment). Because of the occult location at the time of recurrence, tumors are often not detected at late stages when symptoms appear, which makes treatment difficult. Surgery, chemotherapy, or radiotherapy should be selected according to the histological type.

## Author contributions

JXG and YNB wrote the manuscript, and JGZ provided the analysis.

**Conceptualization:** Ying-Na Bao.

**Data curation:** Jia-Xing Guo, Ying-Na Bao.

**Formal analysis:** Jia-Xing Guo, Ying-Na Bao.

**Investigation:** Jian-Guo Zhao.

**Methodology:** Jia-Xing Guo.

**Project administration:** Jia-Xing Guo, Ying-Na Bao.

**Resources:** Ying-Na Bao.

**Software:** Jian-Guo Zhao.

**Visualization:** Jian-Guo Zhao.

**Writing – original draft:** Jia-Xing Guo, Ying-Na Bao.

**Writing – review & editing:** Jia-Xing Guo, Ying-Na Bao.
